# The influence of contextual factors on the physical demands of soccer match-play

**DOI:** 10.5114/biolsport.2026.157995

**Published:** 2026-01-02

**Authors:** Enrique Benéitez-Andrés, Javier Sánchez-Sánchez, Alejandro Rodríguez-Fernández, Mercedes Sánchez-Barba, Daniel Rojas-Valverde, Mario Sánchez

**Affiliations:** 1 Department of Statistics, Faculty of Medicine, University of Salamanca, Salamanca, Spain; 2 Real Valladolid Football Club, Valladolid, Spain; 3 PRENDE Research Group: Planning and Evaluation of Sports Training and Performance. Pontifical University of Salamanca, Spain; 4 VALFIS Research Group, Department of Physical Education and Sports, Institute of Biomedicine (IBIOMED), Universidad de León, 24071 León, Spain; 5 Department of Statistics, Faculty of Medicine, University of Salamanca, Salamanca, Spain; 6 School of Human Movement Sciences and Quality of Life (CIEMHCAVI), National University, Costa Rica

**Keywords:** Soccer, External load, Performance factors, Principal component analysis, Multivariate analysis, Physiological monitoring

## Abstract

This observational longitudinal study examined the impact of six contextual on the match demands of professional soccer players using Principal Component Analysis (PCA). Data were collected from 17 male players across 37 official matches (September 2023–May 2024) of a Tier 3 Spanish team, utilizing 10 Hz GPS units. PCA, combined with hierarchical cluster analysis, effectively reduced the 17 initial external load metrics to eight representative variables, optimizing relevant information and minimizing redundancy inherent in performance monitoring systems. Six contextual factors (match outcome, location, player position, goal difference, halftime score evolution, and competitive block) were analyzed for their influence using ANOVA. Results indicated that player position was the most influential variable (p ≤ 0.001, large effect sizes), significantly affecting all external load metrics, with distinct profiles for forwards, midfielders, and defenders. Specifically, forwards excelled in high-intensity actions; midfielders recorded higher values in medium-intensity distances and decelerations, while defenders consistently exhibited the lowest overall physical demands. Match location significantly influenced high-intensity distance and decelerations (higher at home, small effect sizes). The competitive block showed a significant effect only on low-intensity distance, peaking during intermediate periods, suggesting that effective load management strategies mitigated fluctuations in high-intensity activity. Conversely, match outcome, goal difference, and score difference evolution had no significant effects on most physical demands. This study confirms player position as the predominant contextual factor and highlights the utility of PCA in refining load monitoring. Practical applications include individualized training regimens tailored to positional demands and optimized monitoring strategies for efficient performance analysis.

## INTRODUCTION

Soccer matches demand repeated high-intensity efforts (i.e., running, changes of direction and jumps) combined with periods of low intensity [1]. These efforts integrate conditional, psychological, technical, and tactical components [2–4], making soccer a highly complex and multifactorial sport. On average, a soccer player covers around 11 km per match [5], of which 91% correspond to low-intensity activities (0–14.3 km · h^−1^), 9% to high-intensity activities (14.4–25.1 km · h^−1^), and 0.6% to sprint activities (≥ 25.2 km · h^−1^) [2, 3]. Despite representing a small proportion of the total distance, high-intensity actions appear to be the most decisive for match outcomes [1].

Specifically, players cover ~451 m at high-speed running, ~247 m at very high-speed running, and ~210 m in sprints, accelerating for approximately 1022 m and decelerating for approximately 899 m [5]. However, soccer is a complex sport, and match demands are influenced by additional factors such as the player’s position, the opponent’s level, tactical formation, and the importance of the match, all of which can either decrease or increase a player’s physical output [6–8]. These factors are commonly referred to as contextual variables and are crucial for understanding the multifaceted nature of soccer performance, as they explain the variability in physical output.

Among the widely studied contextual factors are those intrinsic to the player (e.g., age and experience in the sport) and those related to the match flow (e.g., effective playing time). Player position, game location, match outcome, and the level of the opponent being particularly focused upon [9]. There is a consensus in the scientific literature, supported by robust systematic reviews, that player position is the most influential factor on the physical demands of a football match [10]. This hierarchical importance stems from the fact that positional roles are intrinsic, primary determinants of external load—dictating the fundamental movement patterns required by the tactical system—whereas factors like match outcome or location are secondary modulators of this established base load. This is largely due to the specific tactical roles associated with each position, with external and central midfielders typically covering the greatest total distances, while central defenders and forwards tend to cover the least [11].

Regarding match location (home vs. away), evidence remains inconsistent. Some studies found greater physical demands when playing at home, attributing it to the “home advantage” [11–13]. However, other authors have questioned its relevance, finding no significant differences in physical demands between home and away matches [14]. In fact, recent research has even reported higher values in specific external load metrics, such as high-intensity running distance (TD 14–19 km · h^−1^) and very high-intensity running distance (TD 19–25 km · h^−1^) for away matches [15]. Furthermore, some studies suggest that opponent quality and match outcome are contextual factors with a greater influence than match location [16, 17]. It has also been reported that a team’s winning status leads to a decrease in high-intensity distance covered over time [11].

Despite the established literature focusing primarily on the final match outcome (win, loss, or draw) as a determinant of physical load [11, 13], it is essential to consider more granular contextual variables. In this regard, our study adopts a novel approach by analyzing the effect of goal difference and the evolution of the score from halftime to the end of the match. This approach allows us to evaluate whether the game’s dynamics, and not just the result, significantly influence players’ physical demands, offering a more detailed perspective than previous works.

However, most of these studies perform an analysis based on a traditional methodology that presents a challenge in data management due to the difficulty of correctly selecting and interpreting the large number of variables provided by the monitoring systems, which can potentially reach ~100 or ~200 [18]. These variables are often characterized by high multicollinearity, as many of them measure similar aspects of physical load (e.g., total distance is highly correlated with low-intensity distance and medium-intensity distance). This high level of data redundancy and interdependence not only complicates the selection and interpretation of key performance indicators but can also compromise the statistical validity of traditional analyses like ANOVA, making it difficult to establish clear, robust relationships between variables [19]. Therefore, a more robust statistical approach is needed to reduce dimensionality and manage this complexity.

PCA is an analysis technique that aims to identify logical combinations by reducing the dimensionality of a data set composed of highly correlated variables [20]. PCA, when applied to football data, reduces the large number of variables that are managed daily, regardless of the tracking system used for their collection, helping coaches select and interpret key load indicators in the context of each team [21].

Therefore, the primary objective of this study was to investigate the influence of multiple contextual variables (i.e., player position, match location, competitive block and score difference) on match demands in professional soccer players, utilizing Principal Component Analysis (PCA) as a multivariate dimensionality reduction technique to uncover latent patterns in the data. It was hypothesized that the PCA technique offers a better approach to analyzing the impact of contextual variables on match demands, due to its ability to select and interpret only the key load indicators [19].

## MATERIALS AND METHODS

### Design

An observational longitudinal study design was conducted from September 2023 to May 2024, analyzing 37 official matches of a professional soccer team. Matches were played on Saturday or Sunday, depending on the official calendar, meaning the length of the microcycles continually varied. Half of the matches were played at home (n = 19) and the other half were played away (n = 18). The team’s tactical system was based on a consistent 1-4-3-3 formation, as reported by the coaching staff for each of the matches analyzed. This reliance on the club’s internal data is common in this professional context. This approach ensured that player roles and positional demands remained stable across all observations, which is crucial for a robust analysis of contextual variables. Furthermore, the consistency of the formation was indirectly supported by the stability of the positional categorization and the clear delineation of roles observed in the positional tracking data (e.g., center-backs remaining central, full-backs operating wide), confirming the team’s commitment to a specific game model throughout the season. Data were collected using portable GPS devices to record match demands.

### Participants

Data were collected from 17 male professional soccer players (age: 26.47 ± 6.13 years; height: 176.88 ± 6.62 cm; weight: 70.21 ± 7.38 kg; soccer playing experience: 19.47 ± 6.13 years; professional playing experience: 6.81 ± 6.18 years) from a Tier 3, Highly Trained/National Level team (2ª RFEF, Spain) [22]. Players trained 4–6 times per week and completed two gym sessions. Players from different positions were included in the analysis: defenders (D, n = 6), midfielders (M, n = 5), and forwards (F, n = 6). For analysis purposes, wingers were classified as forwards due to their similar tactical roles and high-intensity demands [23]. Players who did not play the entire match and goalkeepers were excluded from the analysis due to the different nature of their activity profiles. The final sample analyzed consisted of 476 individual observations, each corresponding to a half (first or second period) played by a player in the analyzed matches. Thus, for every match, two independent observations per player were registered (one per half), allowing for a more granular analysis of contextual influences across different phases of the game. All procedures were conducted in accordance with the ethical standards of the Declaration of Helsinki. Before data collection, all players provided their written consent and authorization to the club for the use of their routine performance data as part of standard club operations [24]. For the purpose of this research, all collected data were anonymized to ensure player confidentiality. Of the 43 official matches played during the season, 37 were included in the analysis, as six were excluded due to GPS activation errors or tracking failures that resulted in incomplete or invalid data. No imputation procedures were applied, since cases with missing or invalid tracking data were removed prior to analysis.

### Contextual factors

Six independent contextual variables were included in the research in line with previous studies: i) final match outcome, categorized into three levels (win, loss, and draw) [25]; ii) match location (home and away) [12]; iii) player position (defender, midfielders, and forwards) [10]; iv) goal difference at full-time, categorized as minimal difference (± 1 goal), wide favorable difference (≥2 goals), or wide unfavorable difference (≥ 2 goals)) [26]; v) score evolution from half-time to full-time, (no point to no points (NP-NP), no points to points (NP-P), points to no points (P-NP), or points to points (PP)) [27]; vi) competitive block, divided into eight consecutive time blocks according to the competitive calendar These blocks spanned the entire season: (Weeks 1–5, 6–10, 11–15, 16–20, 21–25, 26–30, 31–35, and 36–39) [28]. Interaction effects between contextual factors (e.g., match location × player position) were not analyzed, as further segmentation would have led to very small subgroups and reduced robustness. However, the variable ‘change in result from halftime to full time’ already captures an interaction between two time points within the match.

### External load variables

GPS units initially export 17 match demands variables expressed with total values, counts, and maximum values: (1) TD: total distance covered in meters; (2) TD1: total distance covered in meters from 0 to 7 km · h^−1^; (3) TD2: total distance covered in meters from 7 to 14 km · h^−1^; (4) TD3: total distance covered in meters from 14 to 19 km · h^−1^; (5) TD4: total distance covered in meters from 19 km · h^−1^ to 25 km · h^−1^; (6) TD5: total distance covered in meters over 19 km · h^−1^; (7) TD6: total distance covered in meters over 25 km · h^−1^; (8) SPR: total actions over 25 km · h^−1^; (9) AVG: average velocity in km · h^−1^; (10) V_max_ (km · h^−1^): Maximum speed reached in the match in km · h^−1^; (11) A_max_: Maximum acceleration reached in the match in m · s^−2^; (12) ACC1: total accelerations performed over 2.5 m · s^−2^; (13) ACC2: total accelerations performed over 3 m · s^−2^; (14) ACC3: total accelerations performed over 4 m · s^−2^; (15) DEC1: total decelerations performed over 2.5 m · s^−2^; (16) DEC2: total decelerations performed over 3 m · s^−2^; (17) DEC3: total decelerations performed over 4 m · s^−2^.

To ensure data robustness, a portable 10 Hz GPS unit was utilized (YOOMEDOO sports tracker v3), a frequency widely considered a reliable standard for accurately quantifying the high-intensity actions inherent to soccer [29, 30]. This specific device has been previously utilized in similar soccer contexts to assess match demands [31]. The initial 17 variables exported by the device were selected as they represent the most common and theoretically relevant metrics for describing external load [29]. The specific thresholds utilized for speed and acceleration ranges are similar to those used and reported by previous studies: TD1 (low intensity), TD2 (medium intensity), TD3 (high intensity), TD4 (very high intensity), TD6 (sprint), ACC3, and DEC1 [32, 33]. Strict adherence to manufacturer protocols—including device activation 10 minutes prior to warm-up and the exclusion of matches with incomplete data—minimized positional error. This rigorous approach to data collection and variable selection ensures that the metrics used are valid for the complex demands of professional soccer.

### Procedures

To quantify the match demands, each player wore a portable 10 Hz GPS unit with integrated 100 Hz accelerometers (YOOMEDOO sports tracker v3; Yoomedoo SL, Palma de Mallorca, Spain) attached to the upper back via a specially designed vest. This device has been previously used to assess match demands in soccer players [31]. The devices were activated 10 minutes before each player started the warm-up before the match to ensure adequate satellite reception. During the monitoring period, each player consistently used the same device, which was always fitted and checked by the team’s strength and conditioning trainer [30]. Finally, the data were downloaded through the manufacturer’s platform and processed in an Excel spreadsheet.

### Statistical analysis

Three distinct levels of statistical analysis were applied: i) exploratory descriptive analysis of the match demands variables; ii) calculation of measures of central tendency (mean and median), dispersion (standard deviation [SD], interquartile range [IQR]), and extreme values (minimum and maximum) to fully characterize the distribution of each variable; iii) application of multivariate dimensionality reduction techniques to identify variables with greater explanatory power within the original dataset and reduce informational redundancy. Given the observational and retrospective nature of the study, utilizing a complete season of data from a professional team, a priori sample size calculation was not performed. However, the large number of individual observations (N = 476) is considered sufficient to provide statistical power to detect meaningful effects within this specific population. Dimensionality reduction was performed using Principal Component Analysis (PCA), following the reporting framework proposed by Rojas-Valverde et al. [34], and achieving a full compliance score (21/21). Prior to analysis, variables were scaled and centered, and data suitability was verified through the Kaiser– Meyer–Olkin (KMO) index and Bartlett’s test of sphericity. In the initial PCA, four components with eigenvalues > 1 were identified, of which the first two explained the largest share of variance. Guided by absolute loadings ≥ 0.60, low multicollinearity (Pearson r < 0.75), and conceptual relevance to soccer performance, the dimensionality was reduced to eight representative variables. The final PCA showed good sampling adequacy and significant sphericity, with two components > 1 retained (PC1 and PC2), jointly explaining 63.7% of total variance. Hierarchical clustering and biplot visualizations supported the interpretation, with no rotation applied given the clarity of the factorial structure. This rigorous process yielded eight representative variables that balanced theoretical relevance, practical applicability, and statistical robustness for subsequent analyses. Our analytical approach, based on PCA for dimensionality reduction followed by subsequent one-way ANOVAs, was a deliberate methodological choice. This procedure allowed us to identify representative metrics and independently assess the influence of each contextual variable, which facilitates a clear and practical interpretation for soccer practitioners.

Variables were graphically represented using factorial planes derived from PCA. Additionally, an agglomerative hierarchical cluster analysis was employed, utilizing Euclidean distance and Ward’s method, to visualize the natural grouping of variables based on their statistical proximity via a dendrogram. Subsequently, the influence of the contextual variables (match result, match location, player position, goal difference, change in result from halftime, and competitive block) on the metrics resulting from the dimensionality reduction process was evaluated. This analysis was performed using one-way analysis of variance (ANOVA), with statistical significance determined by an adjusted alpha value of 0.05. Effect sizes were calculated using Partial Eta Squared (η_p_^2^), interpreted according to as follows: η_p_^2^ ≥ 0.14 (large), between 0.06 and 0.13 (moderate), between 0.01 and 0.05 (small), and < 0.01 (very small or trivial) [35]. In cases where ANOVA revealed significant effects, posthoc comparisons were conducted using Tukey’s test, applying correction for multiple comparisons. The magnitude of differences between groups was calculated using Cohen’s d, interpreted according to the following standard criteria [35]: trivial (< 0.20), small (0.20 to 0.49), moderate (0.50 to 0.79), and large (≥ .80). The Standardized Mean Difference (SMD) with a 95% confidence interval (95% CI) was also calculated to complement the quantitative and clinical interpretation of the observed differences. To facilitate a visual and integral interpretation of players’ conditional profiles according to contextual variables, graphical representations were created using biplots derived from PCA, where each observation was projected based on their conditional characteristics and categorized according to the studied contextual variables. All analytical procedures were carried out using the R statistical software (version 4.1.2; R Core Team, 2021), primarily utilizing the FactoMineR, factoextra, ggplot2 and effect size libraries [36, 37]. The level of statistical significance was set at p < 0.05.

## RESULTS

A total of 476 valid observations were analyzed. The distribution of contextual variables (match outcome, player position, match location, competitive blocks, goal difference and score evolution) is presented in Table 1.

**TABLE 1:** Distribution and cross-classification of the contextual variables

Variable / Category / N (%)			Match outcome			Player position			Match location		Goal difference			Score evolution			

			L	D	W	D	M	F	H	A	± 1	≥ 2 (C)	≥ 2 (F)	NP-NP	NP-P	P-NP	P-P
Match outcome	L	111 (23.3)

	D	135 (28.4)

	W	230 (48.3)

Player position	D	191 (40.1)	50 (10.5)	49 (10.3)	92 (19.3)

	M	144 (30.3)	29 (6.1)	44 (9.2)	71 (14.9)

	F	141 (29.6)	32 (6.7)	42 (8.8)	67 (14.1)

Match location	H	241 (50.6)	55 (11.6)	46 (9.7)	140 (29.4)	95 (20.0)	74 (15.5)	72 (15.1)

	A	235 (49.4)	56 (11.8)	89 (18.7)	90 (18.9)	96 (20.2)	70 (14.7)	69 (14.5)

Goal difference	± 1 goal	335 (70.4)	73 (15.3)	135 (28.4)	127 (26.7)	134 (28.2)	101 (21.2)	100 (21.0)	175 (36.8)	160 (33.6)

	≥ 2 (C)	38 (8.0)	38 (8.0)	—	—	17 (3.6)	11 (2.3)	10 (2.1)	—	38 (8.0)

	≥ 2 (F)	103 (21.6)	—	—	103 (21.6)	40 (8.4)	32 (6.7)	31 (6.5)	66 (13.9)	37 (7.8)

Score evolution	NP-NP	58 (12.2)	58 (12.2)	—	—	28 (5.9)	15 (3.2)	15 (3.2)	26 (5.5)	32 (6.7)	32 (6.7)	26 (5.5)	—

	NP-P	63 (13.2)	—	48 (10.1)	15 (3.2)	22 (4.6)	20 (4.2)	21 (4.4)	23 (4.8)	40 (8.4)	63 (13.2)	—	—

	P-NP	53 (11.1)	53 (11.1)	—	—	22 (4.6)	14 (2.9)	17 (3.6)	29 (6.1)	24 (5.0)	41 (8.6)	12 (2.5)	—

	P-P	302 (63.4)	—	87 (18.3)	215 (45.2)	119 (25.0)	95 (20.0)	88 (18.5)	163 (34.2)	139 (29.2)	199 (41.8)	—	103 (21.6)

Competitive blocks	1	64 (13.4)	20 (4.2)	—	44 (9.2)	27 (5.7)	16 (3.4)	21 (4.4)	30 (6.3)	34 (7.1)	35 (7.4)	—	29 (6.1)	6 (1.3)	15 (3.2)	14 (2.9)	29 (6.1)

	2	60 (12.6)	—	—	60 (12.6)	27 (5.7)	16 (3.4)	17 (3.6)	35 (7.4)	25 (5.3)	60 (12.6)	—	—	—	—	—	60 (12.6)

	3	62 (13.0)	24 (5.0)	13 (2.7)	25 (5.3)	26 (5.5)	17 (3.6)	19 (4.0)	25 (5.3)	37 (7.8)	25 (5.3)	12 (2.5)	25 (5.3)	—	—	24 (5.0)	38 (8.0)

	4	51 (10.7)	—	13 (2.7)	38 (8.0)	21 (4.4)	15 (3.2)	15 (3.2)	25 (5.3)	26 (5.5)	39 (8.2)	—	12 (2.5)	—	—	—	51 (10.7)

	5	64 (13.4)	28 (5.9)	23 (4.8)	13 (2.7)	28 (5.9)	18 (3.8)	18 (3.8)	25 (5.3)	39 (8.2)	37 (7.8)	14 (2.9)	13 (2.7)	28 (5.9)	—	—	36 (7.6)

	6	63 (13.2)	12 (2.5)	38 (8.0)	13 (2.7)	22 (4.6)	19 (4.0)	22 (4.6)	37 (7.8)	26 (5.5)	50 (10.5)	—	13 (2.7)	12 (2.5)	13 (2.7)	—	38 (8.0)

	7	62 (13.0)	—	25 (5.3)	37 (7.8)	21 (4.4)	25 (5.3)	16 (3.4)	38 (8.0)	24 (5.0)	51 (10.7)	—	11 (2.3)	—	12 (2.5)	—	50 (10.5)

	8	50 (10.5)	27 (5.7)	23 (4.8)	—	19 (4.0)	18 (3.8)	13 (2.7)	26 (5.3)	24 (5.0)	38 (8.0)	12 (2.5)	—	12 (2.5)	23 (4.8)	15 (3.2)	—

Match result: L = Lost; D = Draw; W = Win. Playing position: D = Defender; M = Midfielder; F = Forward. Match Location: H = Home; A = Away.

Goal Difference: ± 1 = Goal difference of plus or minus 1; ≥2 (C) = Goal difference of 2 or more goals conceded (against our team); ≥ 2 (F) = Goal difference of 2 or more goals scored (in favor of our team).

Match Points Evolution: NP-NP = No Points (halftime) to No Points (full time); NP-P = No Points to Points; P-NP = Points to No Points; P-P = Points to Points.

Competitive blocks: 1 = Week 1 to Week 5; 2 = W6–W10; 3 = W11–W15; 4 = W16–W20; 5 = W21–W25; 6 = W26–W30; 7 = W31–W35; 8 = W36–W39

The initial PCA showed an acceptable sampling adequacy (KMO = 0.617) and significant sphericity (Bartlett p < 0.05). Four components had eigenvalues > 1, with PC1 = 32.5% and PC2 = 20.3%, explaining a cumulative 52.9% of total variance. Guided by loading thresholds (|loading| ≥ 0.60), correlation patterns, and hierarchical clustering, the dimensionality was reduced to eight representative variables. The final PCA demonstrated good sampling adequacy (KMO = 0.708) and significant sphericity (Bartlett p < 0.05), with two components > 1 (PC1 = 34.9%, PC2 = 28.7%, cumulative 63.7%), and no strong cross-loadings detected, confirming the robustness of the reduced structure.

Table 2 displays the match demands analyzed across the 37 professional football matches. The table includes the mean, SD, Mdn, IQR, and range (minimum–maximum) for each variable, providing an overview of the central tendency and variability in the players’ match demands.

**TABLE 2:** Descriptive statistics of the match demands in the variables included in the Principal Component Analysis

Variable		Mean ± SD	Median (IQR)	Range (Min – Max)
Distance (m)	**TD**	9470.1 ± 886.1	9493.3 (1174.1)	7858.0–11888.9

	**TD1**	3844.7 ± 256.2	3857.0 (331.1)	3113.1–4400.1
	**TD2**	3660.1 ± 603.8	3627.0 (816.5)	2551.5–5784.0
	**TD3**	1315.2 ± 372.8	1272.4 (489.1)	693.4–2652.7
	**TD4**	547.2 ± 210.3	526.5 (287.5)	222.7–1204.5
	**TD5**	651.1 ± 259.5	609.3 (357.9)	239.6–1383.8
	**TD6**	103.9 ± 67.7	91.8 (97.7)	15.9–249.0

	**SPR (n)**	8.2 ± 4.7	7.5 (6.8)	0.8–18.1
	**AVG (km · h^−1^)**	9.5 ± 3.2	10.5 (6.2)	5.1–15.9
	**V_max_ (km · h^−1^)**	28.9 ± 1.8	29.0 (2.2)	24.2–33.1
	**A_max_ (m · s^−2^)**	6.8 ± 2.2	8.1 (4.2)	3.8–10.3

**ACC (n)**	**ACC1**	46.8 ± 10.0	46.1 (13.1)	25.4–74.1
	**ACC2**	38.1 ± 10.5	36.6 (14.3)	20.5–66.6
	**ACC3**	6.6 ± 4.5	5.6 (4.5)	0.7–21.8

**DEC (n)**	**DEC1**	40.1 ± 9.6	38.8 (13.2)	21.8–63.9
	**DEC2**	38.0 ± 10.8	36.9 (15.8)	18.7–70.7
	**DEC3**	13.0 ± 6.7	12.1 (7.5)	1.5–34.1

Note: TD = Total distance; TD1 = total distance covered in meters from 0 to 7 km · h^−1^; TD2 = total distance covered in meters from 7 to 14 km · h^−1^; TD3 = total distance covered in meters from 14 to 19 km · h^−1^; TD4 = total distance covered in meters from 19 km · h^−1^ to 25 km · h^−1^; TD5 = total distance covered in meters over 19 km · h^−1^; TD6 = total distance covered in meters over 25 km · h^−1^; SPR: total actions over 25 km · h^−1^; AVG (km · h^−1^) = average velocity in km · h^−1^; V_max_ (km · h^−1^): Maximum speed reached in the match in km · h^−1^; A_max_ (m · s^−2^): Maximum acceleration reached in the match in m · s^−2^; ACC1: total accelerations performed over 2.5 m · s^−2^; ACC2: total accelerations performed over 3 m · s^−2^; ACC3: total accelerations performed over 4 m · s^−2^; DEC1: total decelerations performed over 2.5 m · s^−2^; DEC2: total decelerations performed over 3 m · s^−2^; DEC3: total decelerations performed over 4 m · s^−2^; SD = Standard Deviation; IQR = Interquartile Range; Min = Minimum; Max = Maximum.

**FIG. 1:**
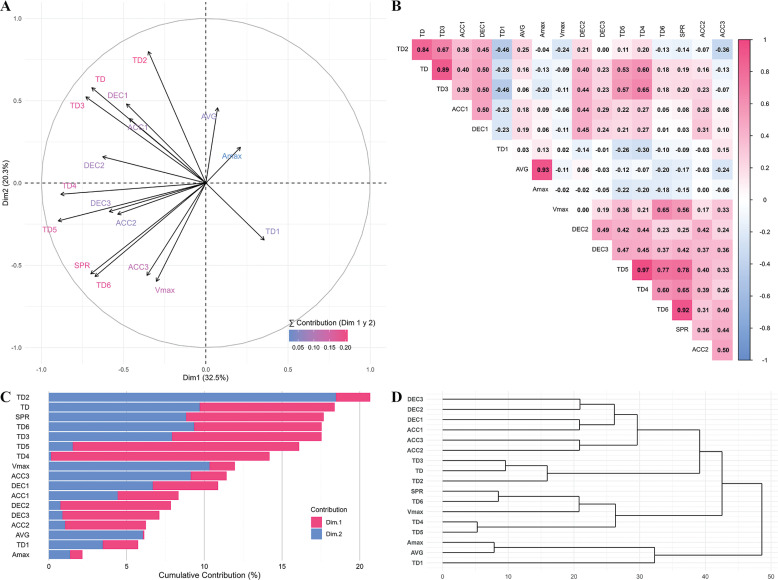
(A) PCA Plot. Variable Projection with Color Gradient by Contribution. (B) Pearson Correlation Matrix with Color Gradient, (C) Cumulative Contributions of the First Two Components, and (D) Hierarchical Clustering Dendrogram. Note: TD = Total distance; TD1 = total distance covered in meters from 0 to 7 km·h^-1^; TD2 = total distance covered in meters from 7 to 14 km·h^-1^; TD3 = total distance covered in meters from 14 to 19 km·h^-1^; TD4 = total distance covered in meters from 19 km·h^-1^ to 25 km·h^-1^; TD5 = total distance covered in meters over 19 km·h^-1^; TD6 = total distance covered in meters over 25 km·h^-1^; SPR: total actions over 25 km·h^-1^; AVG (km·h^-1^) = average velocity in km·h^-1^; Vmax (km·h^-1^): Maximum speed reached in the match in km·h^-1^; Amax (m·s^-2^): Maximum acceleration reached in the match in m·s^-2^; ACC1: total accelerations performed over 2.5 m·s^-2^; ACC2: total accelerations performed over 3 m·s^-2^; ACC3: total accelerations performed over 4 m·s^-2^; DEC1: total decelerations performed over 2.5 m·s^-2^; DEC2: total decelerations performed over 3 m·s^-2^; DEC3: total decelerations performed over 4 m·s^-2^; SD = Standard Deviation; IQR = Interquartile Range.

Initial exploratory analyses are summarized in Figure 1. The PCA factorial plane (Figure 1A) illustrates the contribution of each variable to the first two principal components, facilitating the visualization of their weight in global variability. The Pearson correlation matrix (Figure 1B) revealed interrelationships among metrics, while the dendrogram (Figure 1D) confirmed coherent variable groupings based on common patterns, thereby supporting the interpretation of the observed relationships. Furthermore, the cumulative variance plot (Figure 1C) was fundamental in determining the optimal number of principal components to retain, based on the proportion of explained variance.

**FIG. 2:**
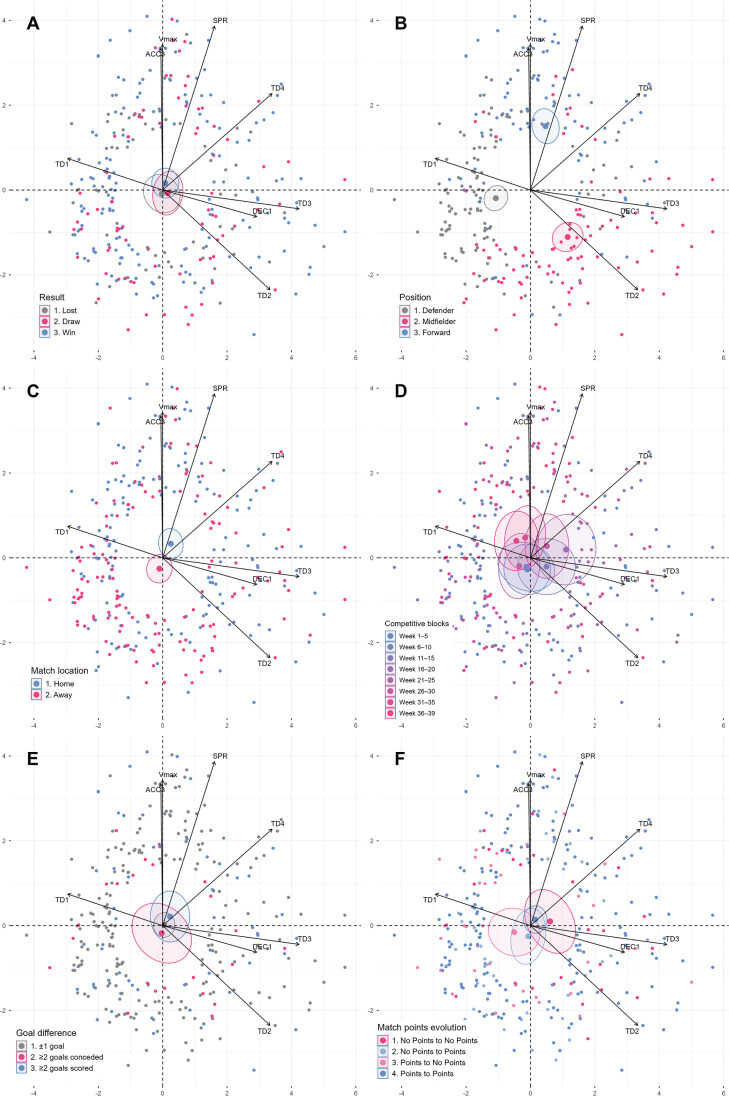
PCA Biplot with Top 300 Contributing Individuals Colored by (A) Result, (B) Position, (C) Match Location, (D) Match Weeks, (E) Goal Difference and (F) Match Points Evolution. Note: TD1 = total distance covered in meters from 0 to 7 km·h^-1^; TD2 = total distance covered in meters from 7 to 14 km·h^-1^; TD3 = total distance covered in meters from 14 to 19 km·h^-1^; TD4 = total distance covered in meters from 19 km·h^-1^ to 25 km·h^-1^; SPR: total actions over 25 km·h^-1^; Vmax: Maximum speed reached in the match in km·h^-1^; ACC3: total accelerations performed over 4 m·s^-2^; DEC1: total decelerations performed over 2.5 m·s^-2^; W = Week; ±1 = Goal differential in a match was plus or minus one. ≥ 2 (C) = Goal difference of 2 or more goals conceded (against our team); ≥ 2 (F) = Goal difference of 2 or more goals scored (in favor of our team).

Table 3 summarizes the inferential analysis of the influence of contextual variables (e.g., match outcome, player position, match location) on the selected external load metrics. Results from the oneway ANOVA and post-hoc comparisons are presented in the table, including statistical significance and effect sizes. Figure 2 provides a visual representation of these findings through a PCA biplot. The specific results for each contextual variable are detailed below.

**TABLE 3:** Inferential Analysis of Contextual Variables on External Load Metrics

	TD1 (m)	TD2 (m)	TD3 (m)	TD4 (m)
**Result**	.514 (NS); .003 (VS) L = D = W	.485 (NS); .003 (VS) L = D = W	.567 (NS); .003 (VS) L = D = W	.819 (NS); .001 (VS) L = D = W

**Position**	.001 (***); .030 (S) F = D > > > M	< .001 (***); .210 (L) M > > > D = F	< .001 (***); .264 (L) M > F > > > D	< .001 (***); .200 (L) F > > M > > D

**Match Location**	.114 (NS); .006 (VS) A = H	.915 (NS); .001 (VS) H = A	.040 (**); .010 (VS) H = A	.021 (**); .012 (S) H > A				

**Competitive blocks**	< .001 (***); .065 (M) 2, 8, 5, 7, 3 > 4, 6 > > 1; 2, 8, 5, 7 > > 6; 2, 8, 5 > > 4 3 > 6; 7 > 4	.065 (*); .031 (S) 3, 6, 4, 2, 1, 7, 5 > 8; 3, 6 > > 8; 4, 2, 1, 7, 5 > 8; 3, 6, 4, 2 > 5; 3, 6, 4 > 7	.110 (NS); .027 (S) 4, 6 > > 5; 3, 8, 7, 2 > 5	.235 (NS); .021 (S) 4 > > 5; 4, 8 > 1; 8, 6, 3, 7, 2 > 5; 4 > 6, 3, 7, 2

**Goal Difference**	.503 (NS); .003 (VS) ≥ 2 (C) = ± 1 = ≥ 2 (F)	.855 (NS); .001 (VS) ≥ 2 (F) = ≥ 2 (C) = ± 1	.957 (NS); .001 (VS) ≥ 2 (F) = ± 1 = ≥ 2 (C)	.759 (NS); .001 (VS) ≥ 2 (F) = ≥ 2 (C)

**Match Points Evolution**	.320 (NS); .008 (VS)tr NP-P, P-P > NP-NP	.100 (NS); .014 (S NP-NP, NP-P, P-P > P-NP	.172 (NS); .012 (S) NP-NP, P-P, NP-P > P-NP	.158 (NS); .012 (S) NP-NP > > P-NP P-P, NP-P > P-NP

**Table:** 

	SPR (n)	V_max_ (km · h−1)	ACC3 (n)	DEC1 (n)
**Result**	.818 (NS); .001 (VS)	.574 (NS); .003 (VS)	.579 (NS); .003 (VS)	.809 (NS); .001 (VS)
	L = D = W	L = D = W	L = D = W	L = D = W

**Position**	< .001 (***); .148 (L)	< .001 (***); .070 (M)	< .001 (***); .267 (M)	< .001 (***); .169 (L)
	F > > > D = M	F = D > > M	F > > > D = M	M > F > > D

**Match Location**	.001 (***); .027 (S)	.236 (NS); .003 (VS)	.236 (NS); .003 (VS)	.001 (***); .023 (S)
	H > A	H = A	H = A	H > A

**Competitive blocks**	.369 (NS); .018 (S) 6, 7 > 5, 2; 6, 7 > 2; 6 > 1	.080 (*); .029 (S) 6 > 7, 2, 4, 8, 3 > 1, 5 6 > > 5; 7, 2, 4, 8, 3 > 5; 6 > > 1; 7, 2, 4, 8, 3 > 1; 6 > 3, 8, 4, 2	.147 (NS); .025 (S) 8, 7 > 6, 4, 1, 5, 3, 2; 8, 7, 6, 4 > 2; 8, 7 > 3, 5, 1, 4	.093 (*); .028 (S) 3, 4, 5 > 8, 7, 1, 6; 3, 4 > 2

**Goal Difference**	.433 (NS); .004 (VS) ≥ 2 (F) = ± 1 = ≥ 2 (C); ≥ 2 (F) > ≥ 2 (C)	.652 (NS); .002 (VS) ≥ 2 (F) = ± 1 = ≥ 2 (C)	.517 (NS); .003 (VS) ≥ 2 (F) = ± 1 = ≥ 2 (C); ≥ 2 (F) > ≥ 2 (C)	.820 (NS); .001 (VS) ≥ 2 (C) = ≥ 2 (F) = ± 1

**Match Points Evolution**	.180 (NS); .011 (S)	.265 (NS); .009 (VS)	.965 (NS); .001 (VS)	.539 (NS); .005 (VS)
	NP-NP, P-P > P-NP	NP-NP, P-P > NP-P	P-P = NP-NP = P-NP = NP-P	NP-NP > P-NP

Note: TD1 = total distance covered in meters from 0 to 7 km · h^−1^; TD2 = total distance covered in meters from 7 to 14 km · h^−1^; TD3 = total distance covered in meters from 14 to 19 km · h^−1^; TD4 = total distance covered in meters from 19 km · h^−1^ to 25 km · h^−1^; SPR: total actions over 25 km · h^−1^; V_max_: Maximum speed reached in the match in km · h^−1^; ACC3: total accelerations performed over 4 m · s^−2^; DEC1: total decelerations performed over 2.5 m · s^−2^; (p-value): NS = Non-significant; * = Marginally Significant (< .10); ** = Significant (< .05); (***) Highly Significant (< .01);

Partial Eta Squared (η_p_^2^): (VS) = Very Small; (S) Small; (M) = Medium; (L) = Large. Post-Hoc (d-cohen): (=) Non-significant; (>) Small; (>>) Moderate; (>>>) Large.

Result: L = Lost; D = Draw; W = Win. Position: D = Defender; M = Midfielder; F = Forward. Match Location: H = Home; A = Away.

Competitive blocks: 1 = Week 1 to Week 5; 2 = W6–W10; 3 = W11–W15; 4 = W16–W20; 5 = W21–W25; 6 = W26–W30; 7 = W31–W35; 8 = W36–W39

Goal Difference: ± 1 = Goal difference of plus or minus 1; ≥ 2 (C) = Goal difference of 2 or more goals conceded (against our team); ≥ 2 (F) = Goal difference of 2 or more goals scored (in favor of our team).

Match Points Evolution: NP-NP = No Points (halftime) to No Points (full time); NP-P = No Points to Points; P-NP = Points to No Points; P-P = Points to Points.

The final match outcome (win, draw, loss) (Figure 2A) did not exert a statistically significant effect on any of the external match demands metrics. Player position (p ≤ .001) (Figure 2B) proved to be a variable with a highly significant influence on all analyzed metrics. Particularly high effect sizes (ES = .200 to .264) were observed in distances covered medium to high intensities (TD2–TD4) and in explosive efforts (ACC3, DEC1). Specifically, forwards excelled in performing high-intensity actions, midfielders recorded higher values in medium-intensity distances and decelerations, while defenders consistently exhibited the lowest physical demands.

Match location (home or away) (Figure 2C) also significantly influenced some metrics, albeit with small effect sizes (ES = .012 to .029). Higher values for high-intensity distance and decelerations were registered when the team played at home. Regarding the competitive block within the season calendar (Figure 2D), only total lowintensity distance (TD1) showed a clearly significant effect, with higher records observed during the intermediate blocks of the season. Despite the presence of certain trends in other variables throughout the season, a consistent temporal pattern was not identified for most external load metrics.

Finally, goal difference (Figure 2E) and the evolution of the score from halftime to the end of the match (Figure 2F) did not reveal statistically significant effects on most external load metrics. Nevertheless, slight trends were detected that might suggest a higher conditional load in matches won by a wide goal difference or when the team maintained or improved its lead in the second half of the match.

## DISCUSSION

The primary objective of the current study was to investigate the influence of contextual variables on match demands in professional soccer players. While our findings confirm that player position is the most influential factor—a pattern well-established in the literature—this study offers significant added value by applying Principal Component Analysis (PCA) as a robust tool to identify and interpret key external load metrics. By reducing data dimensionality and focusing on representative variables, our approach provides a practical framework for load monitoring in a Tier 3 professional context, a competitive level that is under-represented in the scientific literature. Furthermore, this study adopted a novel approach by analyzing more granular contextual factors, such as goal difference and the evolution of the score, to provide a deeper understanding of match dynamics.

The application of Principal Component Analysis (PCA), complemented by a hierarchical cluster analysis, proved crucial in the initial stage of this study. This methodological approach effectively reduced the dimensionality of the data, identifying and selecting eight representative metrics from an initial set of 17. By doing so, it was possible to minimize the redundancy and interdependence among variables, a common challenge in load monitoring. This refinement facilitated a more efficient and robust analysis [18] and enabled a more precise interpretation of key performance indicators [19], which ultimately led to the clear findings on the influence of contextual variables.

Our results indicate that player position is the most influential contextual variable affecting external load, with statistically significant differences observed across all eight metrics identified by the PCA. This influence was particularly strong in medium-to-high intensity running distances and explosive efforts, with large effect sizes observed across these metrics. This highlights the substantial practical relevance of player position on the physical demands of soccer, underscoring the need for highly individualized training programs tailored to each role. These findings align strongly with the existing literature, which consistently identifies player position as the primary determinant of physical demands in soccer [10, 11].

Our methodological choice to group players into three primary positions (defenders, midfielders, and forwards) was made to ensure statistical power and create homogeneous groups for a more robust analysis. However, we recognize that this grouping—specifically the inclusion of Central Defenders and Full-backs within the single ‘Defenders’ group—represents a limitation, as established literature confirms significant differences in their external load profiles [10]. Despite this limitation, the grouping allowed us to maintain the required statistical power for the subsequent analyses and focus on the major performance differences between the three broadest functional roles in soccer. PCA-derived biplots clearly visualize these positional differences, projecting players into the conditional space according to their positional role and confirming the heterogeneity of their external load profiles (e.g., forwards excel in high-intensity actions, midfielders have higher demands in medium-distance and deceleration, and defenders generally have lower load profiles). This can be attributed to the inherent and specific nature of current soccer tactical roles, where each position demands a particular set of actions and a unique distribution of efforts during matches [19]. Forwards are constantly involved in high-intensity sprints and explosive actions (accelerations, sharp decelerations) to get free, press defenders, and seek scoring opportunities, which is reflected in their load profiles, clearly differentiating them from other positions [19]. Midfielders operate in a wider area of the field, requiring them to cover greater total distances and perform many accelerations and decelerations to participate in both defensive and offensive tasks, maintain possession, and organize play, showing high metabolic and neuromuscular demands [38]. Defenders, although they may have lower explosive loads or high-speed distances compared to forwards and midfielders, perform containment actions, individual duels, and changes of direction, implying other specific demands with total load profiles that are often lower in certain metrics [10, 38].

The final match outcome showed no significant effects on match demands. This finding contrasts with previous studies that reported a decrease in distance covered at maximum intensity in winning teams [11]. This discrepancy in results could be attributed to the tactical specifics of the analyzed team, which consistently employed a 1-4-3-3 formation and may prioritize a sustained high-intensity pressing strategy regardless of the score. This suggests that the team’s consistent game model may mitigate the influence of the match outcome on physical output, an aspect that recent studies highlight as a key determinant of match demands [2, 3, 39]. Therefore, our result, rather than contradicting previous work, complements it by demonstrating the crucial role of tactical identity in shaping physical performance. The alternative explanation, that this result is a product of PCA’s ability to select more representative and less redundant metrics, also remains plausible and could be a factor for future studies.

Previous studies have shown that match location (home vs. away) can influence physical performance. In line with previous findings, our results also revealed significantly higher values in specific highintensity metrics, such as high-intensity distance and decelerations, when playing at home [12, 13]. This phenomenon has been associated with increased high-intensity running and decelerations, potentially driven by psychological factors such as crowd support, greater motivation, and familiarity with the environment. This may be attributed to the “home advantage,” a phenomenon associated with psychological factors like crowd support and familiarity with the environment. However, other studies have yielded contradictory results, underscoring the multifactorial and context-dependent nature of home advantage in football [16]. Recent research further suggests that the home factor may not only influence tactical decision-making but also enhance players’ capacity to sustain physically demanding efforts [40]. Despite the statistical significance of our results, the effect sizes observed for match location were small (ηp^2^ = 0.01–0.05), indicating a limited practical impact when compared to other contextual factors.

This phenomenon can be attributed to the constant and dominant influence of tactical demands and inherent positional roles in soccer on physical load, which tends to mitigate, if not override, the more subtle effects of external contextual variables, such as the match venue and match outcome [2, 3, 8]. Positional roles dictate non-negotiable, high-frequency actions (e.g., center-backs must maintain defensive structure, midfielders must shuttle the ball), making these internal tactical requirements a constant ceiling or floor for physical output, regardless of the dynamic, short-term influences of the score or location. However, this dominant influence does not completely nullify the more subtle effects of external contextual variables, such as the match venue. Furthermore, our approach aligns with other recent studies that have successfully used PCA to analyze the influence of contextual factors such as opponent quality on team performance [41].

Regarding the competitive block within the season calendar, a significant effect was observed only in low-intensity distance (TD1), with peaks in the intermediate blocks of the season. This fluctuation, often associated with phases of greater accumulated fatigue or higher training volumes that necessitate more conservative movement on the field [42], contrasts with the lack of a consistent temporal pattern for other higher-intensity variables. This may indicate that the team’s training programs and load management strategies were successful in mitigating fluctuations in high-intensity demands throughout the season. The ability of Principal Component Analysis (PCA) to isolate key variables was fundamental in identifying this specific effect on low-intensity distance, which might otherwise have been diluted in an analysis with a larger number of redundant metrics.

### Study limitations and future directions

This study presents several limitations that should be considered. First, our sample was restricted to a single professional team from a specific league, which limits the generalizability of our findings to other competitive levels, tactical systems, or populations. However, this longitudinal case study offers valuable and in-depth insights into the specific physical demands of a professional team within a competitive context that is under-represented in the scientific literature. Second, the observational design prevents us from establishing causal inferences, and we were unable to control for other influential contextual factors such as opponent quality, match importance, or environmental conditions. This could be a valuable area for future research, as a recent study has successfully applied PCA to analyze the impact of opponent quality and final competition standings on physical performance [41]. Methodologically, the analysis was limited to players who completed the full match, which excludes goalkeepers and substitutes and may not fully represent the ecological demands of the sport. The devices used are 10 Hz GPS units approved by FIFA and widely used in the scientific literature. We acknowledge that this technology has inherent measurement errors, especially for high-speed actions, accelerations, and decelerations. We sought to minimize the impact of these potential positional errors through strict adherence to manufacturer protocols (i.e., device activation 10 minutes prior to warm-up to ensure satellite lock). Our statistical approach, while suitable for our primary objective, did not explore interaction effects between contextual factors due to the risk of creating very small subgroups and reducing statistical power. This could be a consideration for future studies.

### Practical applications

The findings of this study provide relevant insights for coaches, performance analysts, and strength and conditioning staff in professional soccer. Since player position emerged as the main determinant of external load, training programs should be individualized according to positional demands, with forwards requiring greater emphasis on sprinting and explosive actions, midfielders on repeated accelerations and decelerations, and defenders on specific containment and tactical movements. The influence of match location, although small, suggests that playing at home may elicit slightly greater highintensity demands, which should be considered when planning recovery strategies and weekly training loads. Additionally, the effect of the competitive block on low-intensity distances highlights the importance of monitoring accumulated fatigue and adjusting training volumes across the season. Overall, integrating contextual factors such as position, venue, and seasonal phase into load management can help optimize performance, reduce injury risk, and better align physical preparation with the tactical requirements of competition.

## CONCLUSIONS

This study confirms that player position is the primary contextual factor shaping external load in professional soccer, underscoring the need for individualized training programs tailored to positional demands (e.g., sprinting and explosive work for forwards, repeated accelerations and decelerations for midfielders, and tactical containment for defenders). The small influence of match location suggests small adjustments to recovery and training may be warranted after home matches, while the lack of significant effects from match outcome or score evolution indicates that these variables do not meaningfully alter total physical load. Importantly, the use of Principal Component Analysis (PCA) offers practitioners a simplified framework for monitoring, focusing on eight key indicators that reduce redundancy and streamline performance analysis, thereby supporting more efficient decision-making in training load management and tactical preparation.
